# Randomized, Double-Blind, Placebo-Controlled Studies of the Safety and Pharmacokinetics of Single and Multiple Ascending Doses of Eravacycline

**DOI:** 10.1128/AAC.01174-18

**Published:** 2018-10-24

**Authors:** Joseph V. Newman, Jian Zhou, Sergey Izmailyan, Larry Tsai

**Affiliations:** a Tetraphase Pharmaceuticals, Inc., Watertown, Massachusetts, USA

**Keywords:** eravacycline, pharmacokinetics

## Abstract

Eravacycline is a novel, fully synthetic fluorocycline antibiotic with *in vitro* activity against aerobic and anaerobic Gram-positive and Gram-negative pathogens, including multidrug-resistant (MDR) bacteria. The pharmacokinetics (PK), urinary excretion, and safety/tolerability of intravenous (i.v.) eravacycline were evaluated in single- and multiple-ascending-dose studies. Healthy subjects received single i.v. doses of 0.1 to 3 mg/kg of body weight or 10 days of treatment with 0.5 or 1.5 mg/kg every 24 h (q24h) over 30 min, 1.5 mg/kg q24h over 60 min, or 1 mg/kg q12h over 60 min. After single doses, total exposure (the area under the plasma concentration-time curve [AUC]) and the maximum plasma concentrations (*C*_max_) of eravacycline increased in an approximately dose-proportional manner. After multiple doses, steady state was achieved within 5 to 7 days. Accumulation ranged from approximately 7% to 38% with the q24h dosing regimens and was 45% with 1 mg/kg q12h. Eravacycline was generally well tolerated, with dose-related nausea, infusion site effects, and superficial phlebitis that were mild or moderate occurring. These results provide support for the 1-mg/kg q12h regimen used in clinical studies of eravacycline.

## Abstract (precis)

Eravacycline is a novel, fully synthetic fluorocycline antibiotic with *in vitro* activity against aerobic and anaerobic Gram-positive and Gram-negative pathogens, including multidrug-resistant (MDR) bacteria. The pharmacokinetics (PK), urinary excretion, and safety/tolerability of intravenous (i.v.) eravacycline were evaluated in single- and multiple-ascending-dose studies.

## INTRODUCTION

Eravacycline is a novel, fully synthetic fluorocycline antibiotic that retains activity against bacteria with the two major acquired tetracycline-specific resistance mechanisms: ribosomal protection and active drug efflux (1–3). Eravacycline demonstrates potent *in vitro* activity against aerobic and anaerobic Gram-positive and Gram-negative pathogens, including multidrug-resistant (MDR) bacteria, such as carbapenem-resistant Enterobacteriaceae (CRE) and extended-spectrum beta-lactamase-producing Enterobacteriaceae, carbapenem-resistant Acinetobacter baumannii, methicillin-resistant Staphylococcus aureus, and vancomycin-resistant enterococci (4–11). Clinical studies have demonstrated that intravenous (i.v.) eravacycline is effective for treating serious infections in patients with complicated intra-abdominal infections (cIAI) (12, 13), and eravacycline exhibited a tolerability profile that was similar to that of ertapenem.

We report results from single-ascending-dose (SAD) and multiple-ascending-dose (MAD) studies that evaluated the plasma pharmacokinetics (PK), urinary excretion, and safety/tolerability of i.v. eravacycline in healthy subjects.

## RESULTS

### Subject disposition and baseline characteristics.

The baseline characteristics for subjects in both studies are presented in Table 1.

**TABLE 1:** Baseline characteristics for single- and multiple-ascending-dose studies of i.v. eravacycline

Characteristic	Values for patients in:	
	SAD study (*n* = 56)	MAD study (*n* = 32)
Mean (range) age (yr)	28.1 (18–48)	30.8 (19–50)
No. (%) of male subjects	49 (87.5)	31 (96.8)
Mean (range) wt (lb)	176.7 (128.5–239.0)	174.9 (115.5–255.5)
Mean (range) body mass index (kg/m^2^)	25.7 (19.6–31.5)	25.7 (19.3–31.3)
No. (%) of subjects by race		
White	47 (83.9)	29 (90.6)
Asian	3 (5.4)	1 (3.1)
Black or African American	4 (7.1)	1 (3.1)
Other	2 (3.6)	0

### (i) Single-dose study.

Fifty-six subjects (49 males and 7 females) were enrolled in the single-dose study and included in the safety analyses; 14 subjects received placebo, and 42 subjects received eravacycline. Fifty-four (96%) subjects completed the study. Two subjects, one each in the 0.25- and 1-mg/kg-of-body-weight dose groups, discontinued due to adverse events (AEs), in accordance with the contract research organization's influenza response plan. Plasma concentration data from all subjects who received eravacycline were included in the PK analysis.

### (ii) Multiple-dose study.

Thirty-two subjects (31 males, 1 female) were enrolled in the multiple-dose study; 8 subjects received placebo, and 24 subjects received eravacycline. All subjects completed the study; five subjects receiving eravacycline discontinued the study drug due to adverse events. Plasma concentration data from all subjects who received eravacycline were included in the PK analysis.

### Pharmacokinetic results. (i) Single-dose study.

**FIG 1:**
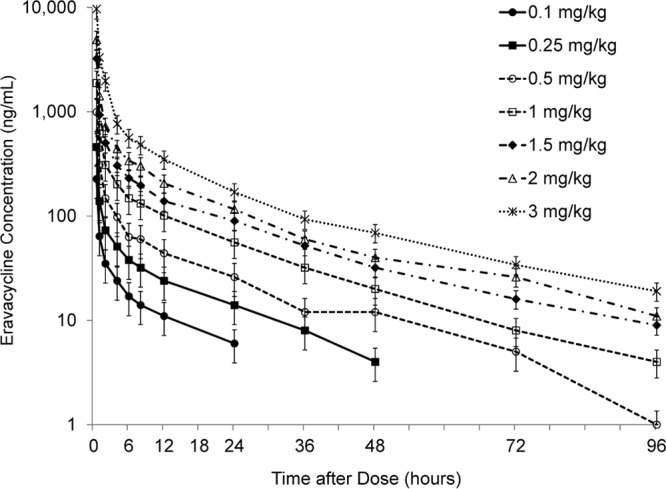
Mean (±SD) plasma concentrations after single i.v. doses of eravacycline.

Plasma concentrations of eravacycline peaked almost immediately after the end of the i.v. infusion and declined in a multiexponential fashion following a single i.v. dose (Fig. 1). The median time to the maximum plasma concentration (*T*_max_) ranged from 0.5 to 0.53 h, and the mean half-life ranged from 12.4 to 24.2 h (Table 2). Dose proportionality was demonstrated for the area under the plasma concentration-time curve (AUC) from 0 h to time *t* (AUC_0–*t*_) over the 0.25- to 3.00-mg/kg dose range. However, maximum plasma concentration (*C*_max_) increased in a more than dose-proportional manner over the 1.00- to 3.00-mg/kg dose range.

**TABLE 2:** PK parameters after single ascending i.v. doses of eravacycline

Parameter^*a*^	Values after the following doses^*b*^:						
	0.1 mg/kg	0.25 mg/kg	0.5 mg/kg	1 mg/kg	1.5 mg/kg	2 mg/kg	3 mg/kg
AUC_0–_*_t_* (ng · h/ml)	428.0 (21.2)	1,153.9 (16.5)	2,502.9 (25.9)	5,297.9 (22.2)	8,495.6 (17.3)	11,972.1 (14.3)	22,443.5 (20.7)
AUC_0–inf_ (ng · h/ml)	554.2 (23.2)	1,309.1 (16.2)	2,678.7 (25.6)	5,511.1 (21.3)	8,856.8 (18.3)	12,347.5 (15.4)	23,145 (20.7)
*C*_max_ (ng/ml)	227.0 (24.5)	466.5 (22.7)	993.5 (17.5)	1,888.3 (27.8)	3,233.3 (22.6)	4,916.7 (17.0)	9,793.3 (16.4)
*T*_max_ (h)	0.5 (0.5–0.6)	0.5 (0.3–0.6)	0.5 (0.5–0.6)	0.5 (0.5–0.7)	0.5 (0.5–0.5)	0.5 (0.5–0.5)	0.5 (0.5–0.6)
*t*_1/2_ (h)	12.7 (23.9)	16.5 (13.3)	19.6 (28.0)	20.2 (28.0)	22.7 (18.0)	22.3 (12.4)	25.6 (9.7)
CL (liter/h/kg)	0.19 (21.3)	0.20 (17.1)	0.20 (24.6)	0.19 (19.8)	0.17 (19.1)	0.17 (14.9)	0.14 (24.5)
*V*_ss_ (liters/kg)	2.5 (14.8)	3.3 (11.6)	3.6 (13.5)	3.4 (20.4)	3.5 (15.4)	3.1 (9.4)	2.3 (26.4)

a AUC_0–_*_t_*, area under the plasma concentration-time curve from 0 h to time *t*; AUC_0–inf_, area under the plasma concentration-time curve from 0 h to infinity; *C*_max_, maximum plasma concentration; *T*_max_, time to maximum plasma concentration; *t*_1/2_, terminal elimination half-life; CL, clearance; *V*_ss_, volume of distribution at steady state.

b Values represent the mean (coefficient of variation [in percent]) for all parameters except *T*_max_, for which the values are the median (range).

**FIG 2:**
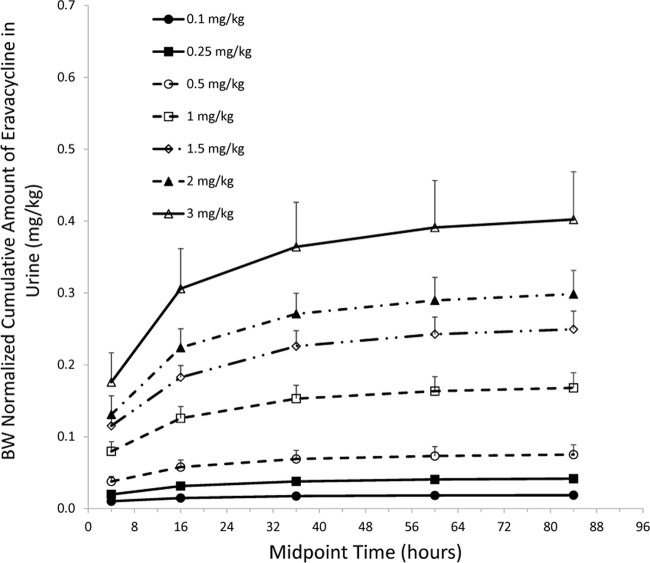
Mean (±SD) body weight (BW)-normalized cumulative urine excretion-time profiles after single ascending i.v. doses of eravacycline in healthy volunteers.

Following a single i.v. dose of eravacycline 0.1 to 3 mg/kg, the cumulative urinary excretion of unchanged eravacycline increased over the 96-h postdose collection period, nearing maximum excretion levels within 24 to 48 h postdose (Fig. 2). Mean cumulative excretion ranged from 1.4 to 32.7 mg, which represented a mean of 13.4% to 18.8% of the administered dose that was recovered in urine.

### (ii) Multiple-dose study.

**FIG 3:**
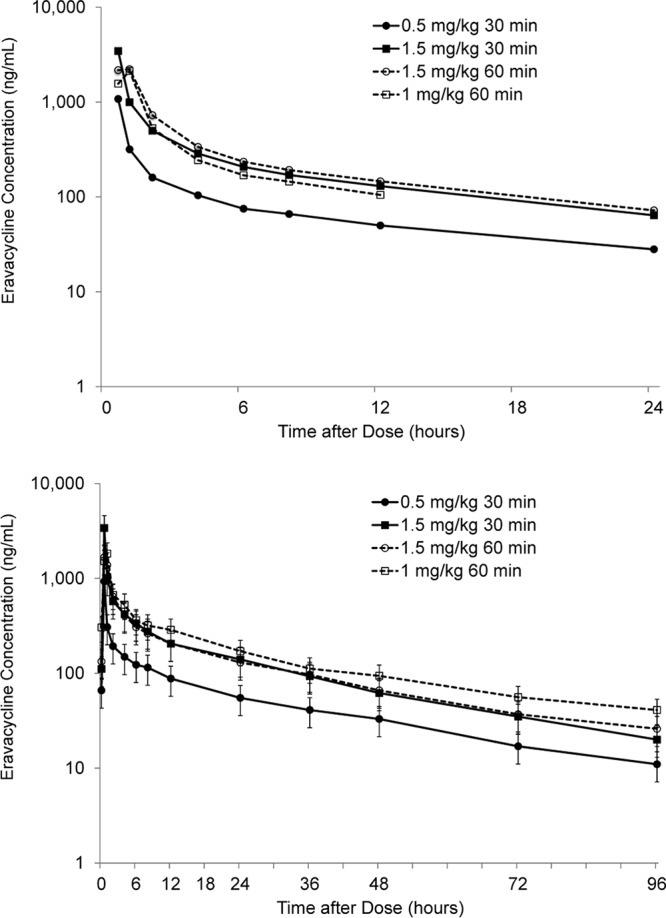
Mean plasma concentrations at day 1 and day 10 after multiple i.v. doses of eravacycline. Values at day 10 are the mean (±SD).

Following the administration of eravacycline at doses ranging from 0.5 to 1.5 mg/kg for 10 days, the mean plasma concentrations on day 1 and day 10 increased with the dose (Fig. 3). The mean peak plasma eravacycline concentrations following the first administration were observed at the end of the infusion, followed by a multiexponential decline. The median *T*_max_ coincided with the end of infusion and was observed at 0.5 h and 1.0 h postdose for the 30- and 60-min infusion dose groups, respectively. The mean *C*_max_ ranged from 1,078 ng/ml to 3,447 ng/ml over the dose range studied. Steady state was reached on days 7, 6, and 5 for the 0.50-mg/kg eravacycline every 24 h (q24h), 1.50-mg/kg eravacycline q24h (30- and 60-min infusions), and 1.00-mg/kg eravacycline every 12 h (q12h) dose levels, respectively. Mean total exposure (AUC) increased with dose, although exposure was impacted by the 30- or 60-min duration of infusion (Table 3). On day 1, dose proportionality was demonstrated for the AUC from 0 to 24 h (AUC_0–24_) and *C*_max_ for eravacycline dose increases from 0.50 to 1.50 mg/kg given q24h over infusion durations of 30 and 60 min. However, the AUC from 0 to 12 h (AUC_0–12_) increases were less than dose proportional for eravacycline dose increases from 1.00 to 1.50 mg/kg. On day 10, dose proportionality was not demonstrated for AUC_0–24_ and *C*_max_ after multiple i.v. infusions of eravacycline over the q24h dose range, with less than dose proportional increases in both parameters being seen after multiple i.v. infusions of eravacycline.

**TABLE 3:** PK parameters after multiple ascending i.v. doses of eravacycline on day 1 and day 10

Day and parameter^*a*^	Values after the following regimens^*b*^:			
	0.5 mg/kg over 30 min q24h	1.5 mg/kg over 30 min q24h	1.5 mg/kg over 60 min q24h	1.0 mg/kg over 60 min q12h
Day 1				
AUC_0–24_ (ng · h/ml)	2,096 (20.9)	6,003 (8.8)	7,171 (15.3)	
AUC_0–12_ (ng · h/ml)				4,305 (13.8)
AUC_inf_ (ng · h/ml)	2,639 (28.5)	7,015 (10.7)	8,385 (15.9)	5,630 (16.1)
*C*_max_ (ng/ml)	1,078 (18.0)	3,447 (7.1)	2,785 (22.0)	2,125 (15.3)
*T*_max_ (h)	0.5 (0.5–0.5)	0.5 (0.5–0.5)	1.0 (1.0–1.0)	1.0 (1.0–1.0)
*t*_1/2_ (h)	12.7 (22.6)	11.0 (11.9)	11.4 (23.4)	8.6 (20.5)
Day 10				
AUC_0–24_ (ng · h/ml)	2,902 (30.4)	8,051 (19.1)	7,592 (11.8)	
AUC_0–12_ (ng · h/ml)				6,309 (15.0)
*C*_max_ (ng/ml)	931 (16.6)	3,403 (9.3)	1,892 (10.3)	1,825 (15.5)
*T*_max_ (h)	0.5 (0.5–0.5)	0.5 (0.5–0.5)	1.0 (0.5–1.0)	1.0 (1.0–1.0)
*t*_1/2_ (h)	35.8 (48.5)	29.1 (14.0)	30.2 (17.0)	38.7 (32.4)
CL (liter/h/kg)	0.19 (31.6)	0.19 (18.2)	0.20 (12.8)	0.16 (17.0)
*V*_ss_ (liter/kg)	5.0 (20.2)	4.0 (18.1)	4.7 (7.7)	4.0 (15.0)

a AUC_0–24_, area under the plasma concentration-time curve from 0 h to 24 h; AUC_0–12_, area under the plasma concentration-time curve from 0 h to 12 h; AUC_inf_, area under the plasma concentration-time curve at infinity; *C*_max_, maximum plasma concentration; *T*_max_, time to maximum plasma concentration; *t*_1/2_, terminal elimination half-life; CL, clearance; *V*_ss_, volume of distribution at steady state. AUC_0–24_ was used for the 24-h regimens, and AUC_0–12_ was used for the 12-h regimen.

b Values represent the mean (coefficient of variation [in percent]) for all parameters except *T*_max_, for which the values are the median (range).

An analysis of PK linearity (the ratio of the AUC from 0 to the dosing interval at steady state [AUC_0-τ_] on day 10 to the AUC from 0 to infinity [AUC_0-∞_] on day 1) and accumulation (the ratio of the AUC_0-τ_ on day 10 to the AUC_0-τ_ on day 1) was undertaken for each dosage regimen (Table 4). The results of this analysis indicated that the AUC_0-∞_ of eravacycline on day 1 and the AUC_0-τ_ on day 10 were similar for each group, a finding which suggests that the PK of eravacycline are linear. Accumulation (by AUC_0-τ_) ranged from approximately 7% to 38% with the q24h dosing regimens and was 45% with 1 mg/kg i.v. q12h.

**TABLE 4:** Analysis of linearity and accumulation of eravacycline across four dosage regimens during multiple-dose administration for 10 days^*a*^

Dose and infusion time	Day 1 mean AUC_0-∞_ (ng · h/ml)	Day 1 mean AUC_0-τ_ (ng · h/ml)	Day 10 mean AUC_0-τ_ (ng · h/ml)	PK linearity ratio of AUC_0-τ_ to AUC_0-∞_	Accumulation ratio of AUC_0-τ_ day 10 to day 1
0.5 mg/kg over 30 min q24h	2,588	2,132	2,932	1.13	1.38
1.5 mg/kg over 30 min q24h	7,129	6,127	7,858	1.10	1.28
1.5 mg/kg over 60 min q24h	8,549	7,340	7,858	0.92	1.07
1.0 mg/kg over 60 min q12h	5,432	4,386	6,344	1.17	1.45

a AUC_inf_, area under the plasma concentration-time curve at infinity; AUC_0-τ_, area under the plasma concentration-time curve from 0 to the dosing interval.

**FIG 4:**
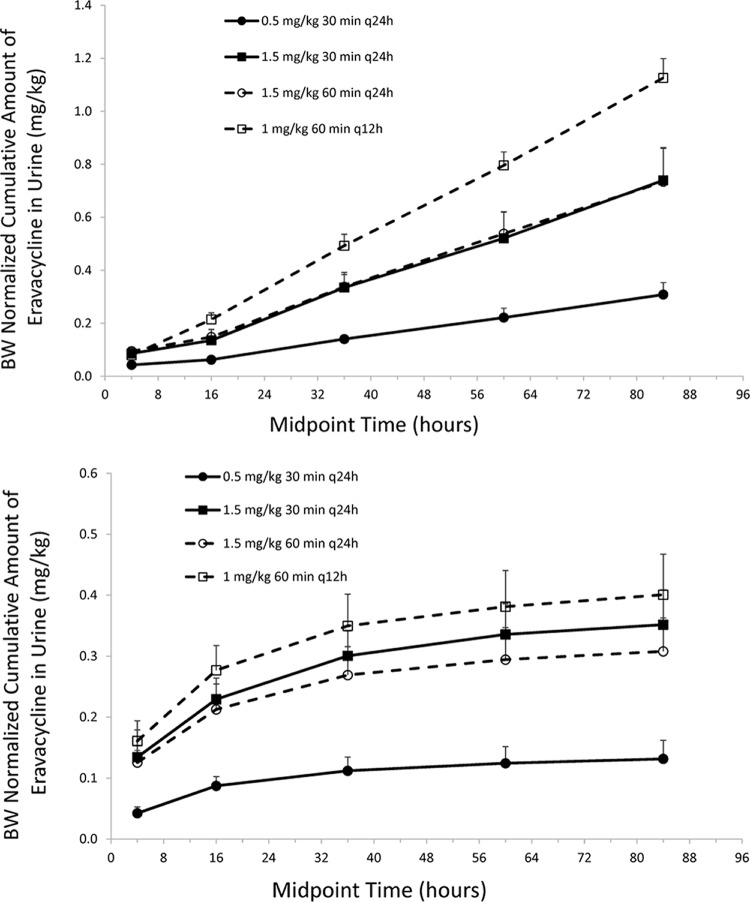
Mean (±SD) body weight-normalized urinary excretion of eravacycline on days 1 to 4 (top) and day 10 (bottom) during the multiple-dose study.

The cumulative urinary excretion of unchanged eravacycline increased over 96 h, nearing maximum excretion levels within 48 to 72 h after the last dose (Fig. 4). The mean cumulative urinary excretion of unchanged eravacycline increased as a function of dose.

### Tolerability.

In the SAD study, adverse events (AEs) occurred in 21.4% of subjects with placebo versus 45.2% of subjects across all doses of eravacycline (Table 5). Treatment-related AEs occurred in 7.1% of subjects with placebo and 31.0% of subjects with eravacycline. All AEs were mild or moderate in severity, and no serious AEs were reported. Two (3.6%) subjects (one each in the 0.25-mg/kg and 1-mg/kg eravacycline groups) discontinued the study for pyrexia as part of an influenza response plan implemented during the influenza season. All episodes of nausea or vomiting occurred at the 2-mg/kg dose (3 episodes of nausea) or 3-mg/kg dose (5 episodes of nausea, 3 episodes of vomiting); all were considered related to eravacycline but were of mild or moderate intensity and were transient. Three subjects experienced infusion site events (pain or phlebitis) that were considered related to treatment. No clinically relevant changes in vital signs, clinical laboratory tests, or electrocardiograms (ECG) were observed. Minor, transient elevations in alanine transaminase (ALT) and aspartate transaminase (AST) levels to approximately 2 times the upper limit of normal (ULN) were observed, but all resolved by the end of the study.

**TABLE 5:** Treatment-emergent adverse events occurring in at least 10% of subjects overall after single and multiple i.v. doses of eravacycline

Adverse event	No. (%) of subjects in:	
	Single-ascending-dose study (*n* = 42)	Multiple-ascending-dose study (*n* = 24)
Any event	19 (45.2)	21 (87.5)
Diarrhea	0	4 (12.5)
Headache	3 (5.4)	6 (18.8)
Infusion site discomfort	0	7 (21.9)
Infusion site pain	2 (3.6)	11 (34.4)
Infusion site erythema	1 (1.8)	10 (41.7)
Nausea	8 (14.3)	11 (45.8)
Pain in extremity	2 (3.6)	5 (15.6)
Phlebitis superficial	0	14 (43.8)

In the MAD study, AEs occurred in 50% of subjects with placebo versus 87.5% of subjects across all 4 dosage regimens of eravacycline, and these were most often dose-related nausea and infusion site reactions (Table 5). Twenty (83.3%) subjects on eravacycline had treatment-related AEs. A dose-related increase in the incidence of AEs was observed, but all AEs were mild or moderate. Nausea occurred in two (33.3%) subjects at 1.5 mg/kg over 30 min, five (83.3%) subjects at 1.5 mg/kg over 60 min, and four (66.7%) subjects at 1 mg/kg q12h over 60 min. Vomiting occurred together with nausea in one subject at 1.5 mg/kg over 30 min and one subject at 1 mg/kg over 60 min. Superficial phlebitis occurred in five (83.3%) subjects at 1.5 mg/kg over 30 min and six (100%) subjects at 1 mg/kg q12h over 60 min. Subjects who received 1.5 mg/kg over 60 min experienced a lower incidence of superficial phlebitis (33.3%). Four subjects (three subjects receiving eravacycline at 1.5 mg/kg over 30 min; one subject receiving eravacycline at 1 mg/kg q12h) experienced superficial phlebitis that resulted in study discontinuation, in addition to one subject who was discontinued for decreased appetite, nausea, and vomiting in the 1-mg/kg q12h group. No subject in the group receiving 1.5 mg/kg over 60 min was discontinued. No serious AEs were reported. Transient elevations in ALT or AST levels to <2 times the ULN, which were not considered clinically significant, occurred in 6 of 24 (25%) subjects with eravacycline and 2 of 8 (25%) with placebo. In these subjects, ALT or AST levels were mildly elevated for 1 or 2 days, except in one subject with an elevation lasting for 15 days. No elevations in serum bilirubin levels were noted. No other clinically relevant changes in vital signs, electrocardiogram (ECG), physical examination, or clinical laboratory tests were observed.

An analysis of nausea and vomiting was conducted to further explore the incidence, duration, and severity in relation to the dosage regimen. Nausea was reported 20 times by 12 subjects (37.5%). All but one of these subjects was randomized to eravacycline. Vomiting together with nausea occurred in two subjects (1.5 mg/kg over 30 min; 1 mg/kg over 60 min). One subject in the 1.0-mg/kg q12h 60-min infusion group was discontinued from the study drug due to decreased appetite, nausea, and vomiting. The duration of nausea events appeared to be longer in the group receiving the highest dose; nausea lasted >5 h in two of seven subjects who received 1.5 mg/kg q24h and in four of four subjects who received 1.0 mg/kg q12h. All events were of mild or moderate severity. Nausea and vomiting events are summarized in more detail in Table 6.

**TABLE 6:** Nausea and vomiting events after single and multiple i.v. doses of eravacycline

Adverse event	Single-ascending-dose study						Multiple-ascending-dose study					
	No. (%) of subjects with the following type of AE:				Median (range) duration (h)	No. (%) of subjects in which AE led to discontinuation of study drug	No. (%) of subjects with the following type of AE:				Median (range) duration (h)	No. (%) of subjects in which AE led to discontinuation of study drug
	Mild	Moderate	Severe	All			Mild	Moderate	Severe	All		
Nausea	8 (19.0)	0	0	8 (19.0)	6 (2.8, 24.7)	0	8 (33.3)	4 (16.7)	0	11^*a*^ (45.8)	4.4 (0.1, 161.0)	1 (4.2)^*b*^
Vomiting	0	3 (7.1)	0	3 (7.1)	0.03 (0.02, 0.03)	0	1 (4.2)	1 (4.2)	0	2 (8.3)	0.05 (0. 0.2)	1 (4.2)^*b*^

a One subject had both mild and moderate events.

a One subject with both nausea and vomiting discontinued study drug prematurely.

## DISCUSSION

Eravacycline demonstrates a broad spectrum of *in vitro* activity against common pathogens, including many MDR pathogens, such as Enterobacteriaceae species and Acinetobacter baumannii (5–9). Because of this potent *in vitro* activity, i.v. eravacycline is undergoing clinical development for treating patients with cIAI. The phase 1 studies with healthy volunteers described here helped to characterize the PK profile of i.v. eravacycline and select doses for testing in patients. The results of these studies were consistent with those of other studies (14, 15).

In these studies, total and peak plasma concentrations after i.v. eravacycline exhibited a dose-proportional increase after both single and multiple doses. The PK profile of eravacycline was generally linear after single doses and exhibited linearity after multiple doses, as evidenced by similar values of AUC_0-∞_ on day 1 and AUC_0-τ_ on day 10 for each of the four dose groups. After multiple i.v. doses given as 30-min i.v. infusions or once-daily or twice-daily 60-min i.v. infusions for 10 days, steady state was achieved within 7 days depending on the dose, and some systemic accumulation was observed at all doses over 10 days, with the greatest accumulation of 45% being observed with the 1-mg/kg q12h regimen from day 1 to day 10. For the 1.0-mg/kg q12h regimen, on day 10 the mean AUC_0–12_ and *C*_max_ values were 4.38 μg · h/ml and 1.83 μg/ml, respectively. These values are similar to those determined as part of a phase 1 bronchopulmonary disposition study, where the mean AUC_0–12_ and *C*_max_ values after seven doses were 4.56 μg · h/ml and 1.29 μg/ml, respectively.

Consistently in both the SAD and MAD studies, the urinary excretion of eravacycline was low, suggesting primarily nonrenal elimination, which was also observed in other studies of eravacycline (15).

The volume of distribution at steady state (*V*_ss_) was approximately 4 liters/kg after multiple doses of i.v. eravacycline, which suggests an extensive tissue distribution. Other studies with i.v. eravacycline have reported a *V*_ss_ of approximately 1.5 liters/kg after a single i.v. dose and approximately 2.6 to 4.2 liters/kg after multiple i.v. doses (14, 15). For other newer tetracyclines, such as tigecycline, the *V*_ss_ in healthy subjects ranged from 6 to 9.1 liters/kg after single and multiple 100-mg i.v. doses (16–18), and for omadacycline it was approximately 2.6 liters/kg after a single 100-mg i.v. dose (19, 20). Thus, these newer compounds exhibit a more extensive tissue distribution than older tetracyclines, as exemplified by tetracycline and doxycycline with volumes of distribution of 1.3 liters/kg and 0.7 liter/kg, respectively (16). The pharmacokinetics and pharmacodynamics of tetracycline-class antibiotics represent a relatively underinvestigated but interesting area of antimicrobial chemotherapy. It is still unclear as to the relative importance of serum versus tissue levels in predicting outcomes, but the distribution in tissue may be important, given the large volumes of distribution of tissue.

Eravacycline was associated with dose-related increases in AEs, mostly nausea, infusion site effects, and superficial phlebitis. However, all AEs were of mild or moderate intensity, with the rate of discontinuation for AEs being low, and no serious AEs were reported. Infusion site AEs occurred in most subjects, although all events were mild or moderate, but infusion site events were more common when i.v. access was via a small peripheral vein. The prolongation of infusion from 30 to 60 min with the concomitant increased dilution of eravacycline was associated with a marked reduction in the incidence of superficial phlebitis. Although minor elevations in ALT and AST occurred, none were clinically significant, and other clinically significant changes in laboratory values, ECG, or vital signs were not observed. This safety/tolerability profile is comparable to that observed in healthy volunteers given i.v. eravacycline at 1 mg/kg q12h for 4 days to assess its bronchopulmonary disposition (14). In phase 2 and 3 studies with i.v. eravacycline at 1 mg/kg infused over 60 min, the rate of nausea was 8% to 11% (12, 13); phlebitis occurred in up to 3.6% of patients, and most events were of mild intensity. Other AEs with i.v. eravacycline occurred at incidences similar to those seen with the comparator, ertapenem.

The occurrence of nausea and vomiting is not unique to eravacycline but occurs commonly with other tetracycline-class antibiotics. For example, in healthy subjects, an examination of the relationship between drug exposure and nausea and vomiting with i.v. tigecycline at 12.5 to 300 mg found that nausea occurred in 38% of subjects and vomiting occurred in 18% of subjects, and 24% and 36% of these events, respectively, were severe (21). In phase 3 studies with tigecycline, the incidence of nausea (20.1%) and vomiting (10.0%) was related to the tigecycline AUC (22). Tigecycline exhibits a linear PK profile, but the incidence of nausea and vomiting with i.v. tigecycline limits the i.v. dose to 100 mg (18, 21, 22).

Overall, the PK results from these two SAD and MAD studies provide support for the 1-mg/kg every 12 hours dosage regimen used in clinical studies of eravacycline for cIAI as the optimal dose for achieving potentially therapeutic plasma concentrations with an acceptable tolerability profile.

## MATERIALS AND METHODS

These studies were conducted in accordance with the International Conference on Harmonisation Guidelines for Good Clinical Practice and the Declaration of Helsinki regarding the treatment of human subjects in a study. The study protocol and consent form were reviewed and approved by the PRACS Institute, Ltd. (Fargo, ND), Institutional Review Board. All subjects provided written informed consent prior to any participation in the study.

### Study design.

Both the single- and multiple-dose studies were randomized, double-blind, placebo-controlled trials conducted at a single center (PRACS Institute, Ltd., Fargo, ND). In each dose group, six subjects were randomized to eravacycline and two were randomized to placebo. Subjects reported to the study site at least 12 h before study day 1 and were confined to the study unit until day 5 in the SAD study and until day 14 in the MAD study. The initial dose of i.v. eravacycline was defined as constituting 10% of the human-equivalent dose of the no observable adverse effect level dose in the most sensitive animal species. For each study, dose escalations were performed sequentially if the postdose safety data for the preceding cohort were acceptable, with the goal of determining the maximum tolerated dose.

### Study treatments.

Subjects were allowed a light breakfast 60 to 90 min before the morning dose of study medication, and no fluids were allowed within 1 h prior to and after drug administration. Eravacycline was supplied in a 30-ml vial containing 50 mg sterile lyophilized powder. The powder was reconstituted with 10 ml of sterile water for injection, which was further diluted prior to administration. In the SAD study, subjects were randomized to placebo or eravacycline administered i.v. at a dose of 0.1, 0.25, 0.5, 1, 1.5, 2, or 3 mg/kg. The study medication was diluted in 5% dextrose in water for injection and infused over 30 min. In the MAD study, subjects were randomized to receive placebo or eravacycline as an i.v. dose of 0.5 or 1.5 mg/kg infused over 30 min every 24 h, 1.5 mg/kg infused over 60 min every 24 h, or 1 mg/kg infused over 60 min every 12 h for a total of 10 days of treatment. For the 30-min infusions, the study medication was diluted in 5% dextrose in water for injection; for the 60-min infusions, the study medication was diluted in 0.9% sodium chloride for injection.

In both studies, maximum-tolerated-dose criteria were used to assess the safety of proceeding to each successive dose level. The presence of 1 or more of the following at the same dose level precluded advancing to the next dose level: (i) ≥2 subjects with ALT levels ≥3 times the ULN, (ii) ≥2 subjects with an absolute reticulocyte count reduced by ≥50% on 2 successive assessments, and (iii) ≥6 subjects with vomiting for at least 5 h (FDA guidance, 2009). In addition, the principal investigator and/or the sponsor were able to decide that a maximum tolerated dose was defined on the basis of a qualitative and quantitative assessment of the available safety and PK data.

### Subject selection.

Healthy male and female subjects were eligible if they were nonsmokers, ages 18 to 50 years, and healthy on the basis of medical history, physical examination, 12-lead ECG, and clinical laboratory testing (hematology, clinical chemistry, coagulation). Women were required to be surgically sterile for at least 6 months prior to screening and to have a negative serum pregnancy test before entering the study.

Subjects were excluded for hypersensitivity to tetracycline or tetracycline derivatives or contraindications to antibiotics; clinically abnormal laboratory values, including ALT, AST, or bilirubin levels more than the ULN; positivity for antibodies against human immunodeficiency virus or hepatitis B or C virus; a history of alcohol or drug abuse or tobacco use; use of any prescription medication within 14 days or over-the-counter medication within 7 days of dosing; or the presence of any medical condition that could interfere with the conduct of the study.

### Study assessments.

In the SAD study, blood samples were obtained within 30 min prior to dosing (0 h); after the start of the i.v. infusion at 15, 30, 35, and 45 min; and at 1, 2, 4, 6, 8, 12, 24, 36, 48, 72, and 96 h. In the MAD study, blood samples were collected within 30 min prior to dosing (0 h) and after the start of the infusion on day 10 at 15, 30, 35, and 45 min and 1, 2, 4, 6, 8, 12, 24, 36, 48, 72, and 96 h. In both studies, urine samples for determination of renal clearance were collected prior to dosing and after the start of the i.v. infusion from 0 to 8 h, 8 to 24 h, 24 to 48 h, 48 to 72 h, and 72 to 96 h. For each time period of urine collection, an aliquot(s) was added to a light-tight container at 5°C until the end of that time period. At the end of each time period of urine collection, the volume was measured, the container was swirled, and 3 1-ml aliquots were removed and placed into prelabeled light-tight vials. The urine vials were frozen in a dry ice-ethanol slurry and placed at −80°C until shipment to the bioanalytical laboratory.

Safety and tolerability were assessed from AEs, physical examination, vital signs (blood pressure, heart rate, respiratory rate, body temperature), ECG, and clinical laboratory tests (hematology, chemistry, and urinalysis).

### Pharmacokinetic analysis.

Eravacycline plasma concentration data were used to calculate the PK parameters by the noncompartmental methodology, including the maximum plasma concentration (*C*_max_), the time to the maximum plasma concentration (*T*_max_), the area under the plasma concentration-time curve (AUC), clearance (CL), the volume of distribution at steady state (*V*_ss_), and the terminal elimination half-life (*t*_1/2_). Pharmacokinetic and pharmacodynamic parameters were calculated using WinNonlin Enterprise (version 5.2) software. Summary tables and/or figures were generated using a validated version of Phoenix WinNonlin (version 6.3) software.

All plasma concentration values below the lower limit of quantification for the assay were treated as missing in the PK analysis, except for those that occurred prior to the first quantifiable concentration on day 1, which were treated as zero. Actual sampling times were used in all PK analyses. Eravacycline plasma and urine concentrations were measured using liquid/liquid extraction followed by liquid chromatography with tandem mass spectrometry/mass spectrometry detection. Eravacycline was validated in the range of 5 to 50 ng/ml, with correlation coefficients of the calibration curves being above 0.99. Mean back-calculated concentrations were between 93.6 and 104% of nominal with coefficients of variation in the range of 3.1 to 7.2% in plasma and 95 to 97.3% of nominal with coefficients of variation of 4.6 to 7.6% in urine. Individual concentrations and PK parameters for eravacycline were summarized with descriptive statistics.
